# Prevalence, Perpetrators, and Patterns of Violence in Pelotas, Brazil: A Cross-Cohort Analysis Over Four Decades

**DOI:** 10.1177/08862605261442583

**Published:** 2026-05-30

**Authors:** Michelle Degli Esposti, Daniel Cequeira, Eduardo Viegas da Silva, Alicia Matijasevich, Iná S. Santos, Helen Gonçalves, Bruna Gonçalves, Aluísio J. D. Barros, Fernando Pires Hartwig, Luciana Tovo-Rodrigues, Fernando C. Wehrmeister, Janaina V. S. Motta, Bernardo L. Horta, Ana M. B. Menezes, Joseph Murray

**Affiliations:** 1 University of Oxford, UK; 2 Instituto de Pesquisa Econômica Aplicada (IPEA), Brasília, Brazil; 3 Universidade Federal de Pelotas, Rio Grande do Sul, Brasil; 4 Universidade de São Paulo, Brasil

**Keywords:** violence, epidemiology, Brazil, gender differences, cross-cohort analysis

## Abstract

Interpersonal violence is a major public health concern in Brazil, yet its epidemiology remains poorly understood. In this study, we examined four decades of violence using the municipality of Pelotas, Brazil, which has uniquely rich administrative and cohort data. We analysed trends in officially recorded and self-reported violence from 1980 to 2024 using administrative data and three population-based birth cohorts (1982, 1993, and 2004). Self-reported violence perpetration was measured using harmonised questionnaires at comparable ages and recall periods. LOESS regression smoothed long-term trends and chi-squared tests assessed changes in perpetrator profiles. Violence increased sharply from the 1980s, peaked in 2017, and declined thereafter, with consistent patterns observed across mortality, hospitalisation, and police records. Cohort data mirrored these trends: 12.6%–15.7% of individuals reported perpetrating violence, with the highest prevalence in the 1993 cohort. Individuals born in 2004 had 21% lower odds of perpetration compared with those born in 1993 (Odds ratio = 0.79; 95% confidence interval [0.69, 0.90]). Physical assault drove these trends, while weapon carriage increased in more recent cohorts. Socio-demographic patterns were largely stable over time; however, female perpetration increased across cohorts, particularly for physical assault and weapon carriage. These findings indicate that the rise in violence, and the more recent decline, in Brazil was universal and affected both lethal and non-lethal forms of violence. Additional research is needed to better understand the recent decline in violence and explore why violence perpetration might be becoming more prevalent among women. Understanding the evolving epidemiology of violence in Brazil is critical for designing responsive violence prevention strategies.

## Introduction

Violence is a leading cause of morbidity and premature mortality in Brazil (Degli Esposti et al., 2023; Reichenheim et al., 2011). The social and economic costs of crime and violence are large, with far-reaching consequences on individual mental health and quality of life and costing Brazilian society over 11% of its gross domestic product (Lopes et al., 2008). In 2024 alone, Brazil recorded over 43,500 homicides – the highest absolute number of violent deaths worldwide (*Our World in Data*, 2026). This high burden reflects a dramatic rise in homicide victimisation, where national rates nearly tripled from the 1980s until recent years (Cerqueira et al., 2025). Although these lethal trends are well documented (Murray et al., 2013), reliance on mortality data has left critical questions unanswered. Has the prevalence of non-lethal violence risen at the same pace as homicide victimisation (van Breen et al., 2023)? Who is committing these acts – have socio-demographic profiles of perpetrators shifted as violence has grown more prevalent? We examine changes in the prevalence and patterns of violence in a Brazilian municipality from 1980 from 2024, using a data triangulation approach that draws on administrative data sources and three consecutive birth cohorts.

## Methods

We analysed changes in officially recorded and self-reported violence from 1980 to 2024, in Pelotas, a city of around 340,000 residents in the southernmost Brazilian state of Rio Grande do Sul, Brazil. Pelotas is home to Brazil’s richest series of population-based birth cohorts, with the first initiated in 1982, and maintains administrative police records dating back to the 1990s, which are not available elsewhere in Brazil. Pelotas’ cohorts include all children delivered in the city’s hospitals from 1 January to the 31 December for the years: 1982 (*n* = 5,914), 1993 (*n* = 5,249), and 2004 (*n* = 4,231). Data were collected using consistent methodology (Gonçalves et al., 2018; Horta et al., 2015; Tovo-Rodrigues et al., 2024).

We measured self-reported perpetration of violence using data from a confidential crime questionnaire, adapted from the Edinburgh Study of Youth Transitions and Crime (McAra & McVie, 2010), which was administered at various ages with multiple recall periods (Supplemental Table S1 and Supplemental Figures S1–S2). We harmonised the age of data collection, recall period, and questionnaire items, analysing lifetime prevalence at 40 years for the 1982 cohort and at 30 years for the 1993 cohort, and past year prevalences at 18 years in the 1993 and 2004 cohorts. We focused on four common measures of violence: physical assault, robbery, and weapon carriage and use (Supplemental Table S1). All responses were coded on a dichotomous scale (“no” vs. “yes”), and any violent crime was defined as responding “yes” at least one of the four question items. Cohort prevalence was disaggregated by consistently collected sociodemographic characteristics of sex (male vs. female), skin colour (White vs. Black/mixed), family income (poorest [Q1-Q2] vs. richest quintiles [Q3-Q5]), and maternal education (0–8 years vs. ≥9 years schooling years), measured at birth (Supplemental Table S2). We also drew on multiple sources of official records to measure violence yearly from 1980 to 2024. This included routinely collected health (mortality and hospitalisations due to physical assault) and police-recorded (lethal crimes and robbery) data (Supplemental Table S1). We derived rates per 100,000 person-years for the entire population of Pelotas.

We visualised changes over time, using LOESS regression to smooth long-term trends, and linked trends to changes in cohort prevalence, matching on calendar year. We conducted simple cross-cohort comparisons to examine change over time, using logistic regression to model overall changes in prevalence and chi-squared tests (or Fisher’s exact test when cell counts were less than 5) to examine changes in the socio-demographic profile of violence perpetrators.

## Results

**Figure 1.**
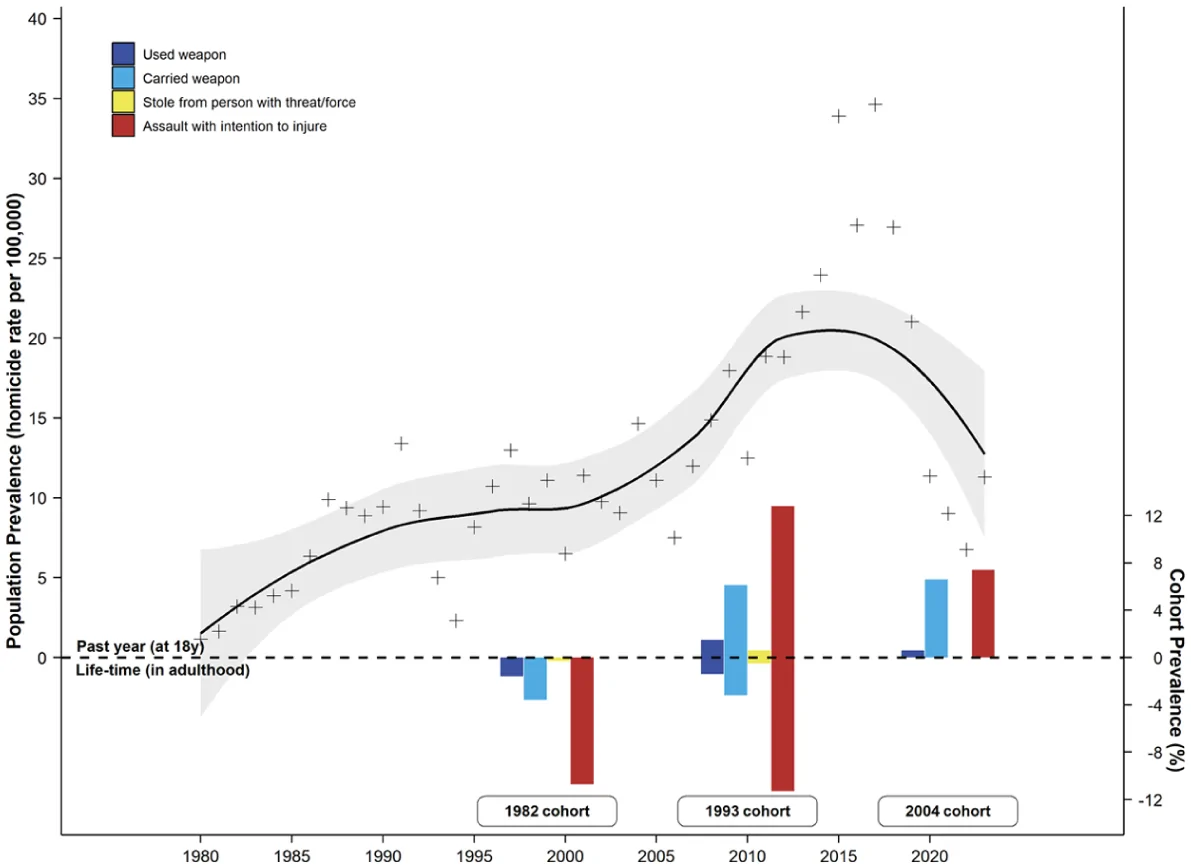
Official records (population prevalence, rates per 100,000) and self-reports (cohort prevalence, rates per 100) of violence in Pelotas, 1980 to 2023. *Note.* Population prevalence is estimated by yearly data on external causes by another person of death (assault, ICD-10: X85-Y09) collected by the national mortality information system (Sistema de Informação sobre Mortalidade, SIM), 1980 to 2023, while cohort prevalence is estimated by comparable self-reported measures of violence perpetration from the 1982, 1993, and 2004 Pelotas Birth Cohort Studies. Data sources were matched by calendar year when cohort members were aged 18 years. Trends were smoothed using LOESS regression with grey ribbons reflecting 95% confidence intervals.

Violence in Pelotas rose sharply from the 1980s, peaking in 2017 and then sharply decreasing until 2024, these patterns broadly mirror national trends across Brazil (Supplemental Figure S3). The increase was not confined to a specific subtype of violence (e.g. lethal violence) or data source (e.g. health-recorded mortality), underscoring the pervasiveness of this pattern (Supplemental Figure S4). Across cohorts, recall periods, and ages, around one in eight persons reported perpetrating violence (12.6%–15.7%; Table 1). The 1993 cohort, which reached peak offending age during the period of highest population rates of violence, showed the highest prevalence of violence behaviour (Figure 1 and Supplemental Figure S5). This pattern is evident when comparing matched age windows (0–17, 18–22, and 23–30 years), between the 1982 and 1993 cohorts, but it was statistically significant only when comparing the 1993 and 2004 cohorts: individuals born in 2004 had 21% lower odds of perpetrating violence than those born in 1993 (odds ratio = 0.79; 95% confidence interval [0.69, 0.90]; *p* < .001, see Table 1).

**Table 1.** Self-Reported Cohort Prevalence of Violence Perpetration Across the 1982, 1993, and 2004 Pelotas Birth Cohort Studies.

	Life-time prevalence^a^					Past year prevalence^b^				
	1982 cohort		1993 cohort		1982 versus 1993 cohorts^c^	1993 cohort		2004 cohort		1993 versus 2004 cohorts^c^
Measure	Total sample (*n*)	% (*n*)	Total sample (*n*)	% (*n*)	ORs (95% CIs)	Total sample (*n*)	% (*n*)	Total sample (*n*)	% (*n*)	ORs (95% CIs)
Any violence	2,938	12.6 (370)	3,224	13.0 (419)	1.04 [0.89, 1.20]	3,632	15.7 (571)	3,221	12.8 (413)	0.79 [0.69, 0.90]***
Sex					0.016					0.001
Female	1,603	31.6 (117)	1,799	40.1 (168)		1,853	29.2 (167)	1,586	39.2 (162)	
Male	1,335	68.4 (253)	1,425	59.9 (251)		1,779	70.8 (404)	1,635	60.8 (251)	
Skin colour					0.357					0.142
Black/mixed	511	19.7 (73)	764	22.7 (95)		829	27.0 (154)	858	31.5 (130)	
White	2,427	80.3 (297)	2,460	77.3 (324)		2,801	73.0 (417)	2,363	68.5 (283)	
Family income					0.918					0.179
Not poor (Q3–5)	1,848	61.9 (229)	1,835	61.3 (250)		2,048	49.9 (279)	1,971	54.5 (225)	
Poor (Q1–2)	1,090	38.1 (141)	1,334	38.7 (158)		1,518	50.1 (280)	1,250	45.5 (188)	
Maternal education					0.208					<0.001
0–8 years	2,133	72.7 (269)	2,375	68.3 (285)		2,709	78.8 (449)	1,752	63.3 (257)	
9 years+	802	27.3 (101)	843	31.7 (132)		917	21.2 (121)	1,439	36.7 (149)	
Assault with intention to injure	2,979	10.7 (319)	3,224	11.3 (369)	1.06 [0.91, 1.24]	3,706	12.8 (476)	3,480	7.4 (259)	0.55 [0.46, 0.64]***
Sex					0.04					0.13
Female	1,625	33.5 (107)	1,799	41.5 (153)		1,884	30.9 (147)	1,699	36.7 (95)	
Male	1,354	66.5 (212)	1,425	58.5 (216)		1,822	69.1 (329)	1,781	63.3 (164)	
Skin colour					0.375					0.021
Black/mixed	523	19.4 (62)	764	22.5 (83)		853	28.2 (134)	937	36.7 (95)	
White	2,456	80.6 (257)	2,460	77.5 (286)		2,851	71.8 (342)	2,543	63.3 (164)	
Family income					0.605					0.873
Not poor (Q3–5)	1,869	62.7 (200)	1,835	64.9 (233)		2,086	50.4 (235)	2,113	51.4 (133)	
Poor (Q1–2)	1,110	37.3 (119)	1,334	35.1 (126)		1,553	49.6 (231)	1,367	48.6 (126)	
Maternal education					0.108					0.008
0–8 years	2,160	71.2 (227)	2,375	65.1 (239)		2,769	78.1 (371)	1,906	68.9 (177)	
9 years+	815	28.8 (92)	843	34.9 (128)		931	21.9 (104)	1,540	31.1 (80)	
Stole from person with threat/force	2,973	0.3 (10)	3,264	0.5 (15)	1.37 [0.62, 3.15]	3,683	0.6 (23)		0.0 (<5)	0.05 [0.00, 0.23]**
Sex					0.626					>0.999
Female	1,622	10.0 (<5)	1,827	20.0 (<5)		1,877	8.7 (<5)	1,588	0.0 (<5)	
Male	1,351	90.0 (9)	1,437	80.0 (12)		1,806	91.3 (21)	1,637	100.0 (<5)	
Skin colour					0.653					>0.999
Black/mixed	521	30.0 (<5)	774	20.0 (<5)		847	30.4 (7)	858	0.0 (<5)	
White	2,452	70.0 (7)	2,489	80.0 (12)		2,834	69.6 (16)	2,367	100.0 (<5)	
Family income					0.428					0.348
Not poor (Q3–5)	1,866	60.0 (6)	1,856	40.0 (6)		2,073	31.8 (7)	1,972	100.0 (<5)	
Poor (Q1–2)	1,107	40.0 (4)	1,352	60.0 (9)		1,543	68.2 (15)	1,253	0.0 (<5)	
Maternal education					0.653					>0.999
0–8 years	2,159	70.0 (7)	2,408	80.0 (12)		2,754	82.6 (19)	1,754	100.0 (<5)	
9 years+	811	30.0 (<5)	850	20.0 (<5)		923	17.4 (<5)	1,440	0.0 (<5)	
Carried weapon	2,986	3.6 (108)	3,270	3.2 (106)	0.89 [0.68, 1.17]	3,700	6.1 (226)	3,479	6.6 (228)	1.08 [0.89, 1.30]
Sex					0.022					<0.001
Female	1,626	17.6 (19)	1,832	32.1 (34)		1,882	17.7 (40)	1,699	39.5 (90)	
Male	1,360	82.4 (89)	1,438	67.9 (72)		1,818	82.3 (186)	1,780	60.5 (138)	
Skin colour					0.575					0.954
Black/mixed	523	21.3 (23)	779	25.5 (27)		849	23.5 (53)	937	24.1 (55)	
White	2,463	78.7 (85)	2,491	74.5 (79)		2,849	76.5 (173)	2,542	75.9 (173)	
Family income					0.057					0.068
Not poor (Q3–5)	1,877	62.0 (67)	1,857	48.1 (50)		2,083	50.2 (112)	2,112	59.2 (135)	
Poor (Q1–2)	1,109	38.0 (41)	1,357	51.9 (54)		1,550	49.8 (111)	1,367	40.8 (93)	
Maternal education					0.944					<0.001
0–8 years	2,167	79.6 (86)	2,411	81.0 (85)		2,763	76.0 (171)	1,905	60.6 (134)	
9 years+	815	20.4 (22)	853	19.0 (20)		931	24.0 (54)	1,539	39.4 (87)	
Used weapon	2,976	1.6 (47)	3,267	1.4 (47)	0.91 [0.60, 1.37]	3,663	1.5 (56)	3,482	0.6 (20)	0.37 [0.22, 0.61]***
Sex					0.592					0.231
Female	1,623	14.9 (7)	1,828	21.3 (10)		1,861	8.9 (5)	1,700	20.0 (<5)	
Male	1,353	85.1 (40)	1,439	78.7 (37)		1,802	91.1 (51)	1,782	80.0 (16)	
Skin colour					0.465					>0.999
Black/mixed	522	27.7 (13)	775	19.1 (9)		836	37.5 (21)	937	40.0 (8)	
White	2,454	72.3 (34)	2,491	80.9 (38)		2,825	62.5 (35)	2,545	60.0 (12)	
Family income					0.535					0.109
Not poor (Q3–5)	1,869	51.1 (24)	1,858	42.6 (20)		2,063	41.1 (23)	2,113	20.0 (<5)	
Poor (Q1–2)	1,107	48.9 (23)	1,352	57.4 (27)		1,533	58.9 (33)	1,369	80.0 (16)	
Maternal education					0.784					0.328
0–8 years	2,162	85.1 (40)	2,411	80.9 (38)		2,732	78.2 (43)	1,907	90.0 (18)	
9 years+	810	14.9 (7)	850	19.1 (9)		925	21.8 (12)	1,540	10.0 (<5)	

*Note.* Counts of less than 5 (*n* < 5) were suppressed.

a Life-time cohort prevalence reflects self-report measures of life-time prevalence measured during mid-adulthood (at 40 and 30 years in the 1982 and 1993 cohorts, respectively) and is quantified as the percent (or rate per 100 persons) of complete cases in each cohort who responded “yes” to violence perpetration.

b Past year cohort prevalence reflects self-report measures of past year prevalence measured at 18 years (in the 1993 and 2004 cohorts) and is quantified as the percent (or rate per 100 persons) of complete cases in each cohort who responded “yes” to violence perpetration. Counts are presented in brackets.

c Cohorts were compared in two ways. To test an overall change in prevalence between the 1982 and 1993 cohort, logistic regression models were fitted. Differences in overall question items therefore reflect odds ratios and corresponding 95% confidence intervals. Specifically, the odds of reporting violence preparation in the 1993 cohort compared to the 1982 cohort. **p < .01; ***p < .001. To test for cohort differences in the sociodemographic profile of offenders, chi-squared tests, or Fisher’s exact test when cell counts were less than 5, were performed. The *p-*values of these tests are reported.

These changes in cohort prevalence were similar across types of violence. Physical assault, the most common form of violence (7.4%–12.8%), drove overall changes over time: slightly increasing between the 1982 and 1993 cohorts and then nearly halving between the 1993 and 2004 cohorts (Table 1). Self-reported robbery and weapon use were rare but followed a similar inverted U-shaped pattern across cohorts – mirroring trends in administrative data. Weapon carriage, in contrast, bucked this recent trend, increasing across the 1993 and 2004 cohorts (Figure 1).

When examining cohort differences by sociodemographic profile, there were few significant patterns (Table 1). One notable exception was perpetration by females – females became significantly more likely to report across cohorts and over time. This was due to rises in physical assault and weapon carriage between the 1982 and 1993 cohorts and again between the 1993 and 2004 cohorts, resulting in a steady increase in the proportion of females committing violence over time. The profile of perpetrators of physical assault also shifted by skin colour and maternal education between the 1993 and 2004 cohorts. However, unlike the observed increases in violence perpetration among females, these patterns may reflect broader changes in the sociodemographic composition of the 2004 cohort (Table 1).

## Discussion

By triangulating across multiple administrative and cohort data sources, we show that the increase in violence in Pelotas from the 1980s up until 2017 was pervasive. The rise cannot be explained by measurement bias from any given data source and was not confined to a specific subtype of violence. Instead, both lethal and non-lethal violence, manifest in victimisation and perpetration data, increased sharply during the critical period. Since 2017, however, population rates of violence have been falling, pointing to an encouraging reversal in a decades-long trend. These promising reductions seem to be best explained by local-level municipal-level initiatives rather than national policies, including the launch of the flagship city-wide violence prevention strategy of Pelotas’ Peace Pact (Degli Esposti et al., 2023). To sustain these declines, policies will need to balance universal prevention strategies with more local efforts that address the changing epidemiology of violence throughout Brazil.

The sociodemographic profile of perpetrators of violence remained largely stable across cohorts, pointing towards a universal, population-wide increase rather than one concentrated among specific subgroups. The marked exception was gender: women in successive cohorts were more likely to report physically assaulting another person and carrying a weapon. This pattern runs counter to existing gender differences in Brazil; where women, compared to men, are 5 times less likely to be victims of physical assault (Gawryszewski & Rodrigues, 2006), 10 times less likely to be victims of homicide (Reichenheim et al., 2011), and more than 10 times less likely to be incarcerated (Murray et al., 2013). But, the change does coincide with a period of profound social and demographic transformation in Brazil, marked by expanding legal and illegal labour markets and a sharp rise in women’s participation (Madalozzo, 2012). These changes reflect a wider, and rapid, move to urbanisation across Brazil, where traditional patriarchal norms that historically constrained women’s role in society gradually started to erode (McIlwaine et al., 2020). Whether our findings reflect a true rise in female-perpetrated violence, or a greater willingness to self-report such behaviour, has important implications for understanding Brazil’s changing epidemiology of violence, with critical implications for targeted prevention strategies.

Our analysis needs to be interpreted with some caution; changes in the socio-demographic profiles of violence perpetrators in Pelotas may not generalise across the rest of Brazil. Despite this, are findings are consistent with previous evidence documenting dramatic changes in population rates of violence in Brazil over the last four decades (Cerqueira et al., 2025). By leveraging uniquely rich municipal-level data, we illustrate how these long-term trends can be unpacked to provide new insights into the prevalence, perpetrators, and patterns of violence at the population level. Future research is needed to better understand the changing epidemiology of violence, including the recent decline, and explore why violence perpetration might be becoming more prevalent among women.

## Supplemental Material

sj-docx-1-jiv-10.1177_08862605261442583 – Supplemental material for Prevalence, Perpetrators, and Patterns of Violence in Pelotas, Brazil: A Cross-Cohort Analysis Over Four Decades Supplemental material, sj-docx-1-jiv-10.1177_08862605261442583 for Prevalence, Perpetrators, and Patterns of Violence in Pelotas, Brazil: A Cross-Cohort Analysis Over Four Decades by Michelle Degli Esposti, Daniel Cequeira, Eduardo Viegas da Silva, Alicia Matijasevich, Iná S. Santos, Helen Gonçalves, Bruna Gonçalves, Aluísio J. D. Barros, Fernando Pires Hartwig, Luciana Tovo-Rodrigues, Fernando C. Wehrmeister, Janaina V. S. Motta, Bernardo L. Horta, Ana M. B. Menezes and Joseph Murray in Journal of Interpersonal Violence
