# Supplementary material for: Prevalence, Perpetrators, and Patterns of Violence in Pelotas, Brazil: A Cross-Cohort Analysis Over Four Decades
Source: J Interpers Violence. 2026 May 30;41(19-20):8354–67. doi: 10.1177/08862605261442583 (PMC13570986; doi:10.1177/08862605261442583)
Supplement: Supplementary file 1 [file sj-docx-1-jiv-10.1177_08862605261442583.docx]

**Prevalence, Perpetrators, and Patterns of Violence in Pelotas, Brazil: A Cross-Cohort Analysis Over Four Decades**

**SUPPLEMENTARY MATERIAL**

| **CONTENTS** | | | **PAGE** |
| --- | --- | --- | --- |
|  | **Supplemental tables** | |  |
|  |  | **Table S1.** Summary of primary data sources and measures of violence. | 2 |
|  |  | **Table S2.** Measures of sociodemographic characteristics consistently collected in the 1982, 1993 and 2004 Pelotas Birth Cohorts. | 5 |
|  | **Supplemental figures** | | |
|  |  | **Figure S1.** Timeline of self-reported violent crime data in the 1982, 1993 and 2004 Pelotas Birth Cohorts. | 6 |
|  |  | **Figure S2.** Overlapping and well-matched measures of self-reported violence in the 1982, 1993 and 2004 Pelotas Birth Cohorts. | 7 |
|  |  | **Figure S3.** Homicide victimization rates per 100,000 person-years in Brazil, State of Rio Grande do Sul, and Municipality of Pelotas, 1980-2023. | 8 |
|  |  | **Figure S4.** Official records of violence rates per 100,000 person-years in Pelotas, 1980-2024. | 9 |
|  |  | **Figure S5**. Self-reported prevalence of violence perpetration at matched age windows of year of last offence (0-17, 18-22, 23-30, 31-40 years) in the 1982 and 1993 Pelotas Birth Cohort Studies. | 10 |
|  | **Supplemental references** | | 11 |

**Table S1.** Summary of primary data sources and measures of violence.

| **Data source** | | | **Measure** | **Unit of analysis** | **Time period**  **(intervals)** | **Age at data collection**  **(recall period)** |
| --- | --- | --- | --- | --- | --- | --- |
| **Officially recorded violence** | | | | | | |
|  | **Police-recorded crime, Secretary for Public Security**^2^ | | | | | |
|  | | Crimes against the person | Lethal violence (Intentional Lethal Violent Crimes, CVLI) | Person (victim) | 1994 – 2023 (yearly) | … |
|  |  | Crimes against property | Robbery (includes violence or threat of violence) | Occurrence (event) | 1993 – 2024 (yearly) | … |
|  | **Health data, Brazilian Ministry of Health**^1,3^ | | | | |  |
|  |  | Mortality due to external causes by another person | Mortality due to assault (ICD-10: X85-Y09) | Person (victim) | 1980 – 2023 (yearly) | … |
|  |  | Hospitalisation due to external causes by another person | Hospitalisations due to assault (ICD-10: X85-Y09) |  | 2014 – 2024(yearly) | … |
| **Self-reported violence** | | | | | |  |
|  | **1982 Pelotas birth cohort**^4^ | | | | |  |
|  |  | Confidential crime questionnaire, adapted from the Edinburgh Study of Youth Transitions and Crime^5^ | Physical assault (“hit people with the intention of hurting them”) | Person (perpetrator) | 1982 – 2022 (40 years) | Aged 40 years old (life-time prevalence, plus age at last offence [0-17y, 18-22y, 23-30y, 31-40y]) |
|  |  |  | Robbery “made threats or used violence in  robbery”) |  |  |  |
|  |  |  | Weapon carriage (“carried a knife or gun for protection or fighting”) |  |  |  |
|  |  |  | Weapon use (“used a weapon against another person”) |  |  |  |
|  | **1993 Pelotas birth cohort**^6^ | | | | | |
|  |  | Confidential crime questionnaire, adapted from the Edinburgh Study of Youth Transitions and Crime^5^ | Physical assault (“hit people with the intention of hurting them”) | Person (perpetrator) | 1993 – 2023 (30 years) | Aged 30 years old (life-time prevalence, plus age at last offence [0-17y, 18-22y, 23-30y]) |
|  |  |  | Robbery “made threats or used violence in  robbery”) |  |  |  |
|  |  |  | Weapon carriage (“carried a knife or gun for protection or fighting”) |  |  |  |
|  |  |  | Weapon use (“used a weapon against another person”) |  |  |  |
|  |  |  | Physical assault (“hit people with the intention of hurting them”) | Person (perpetrator) | 2010 – 2011 (1 year) | Aged 30 years old (life-time prevalence, plus age at last offence [0-17y, 18-22y, 23-30y]) |
|  |  |  | Robbery “made threats or used violence in  robbery”) |  |  |  |
|  |  |  | Weapon carriage (“carried a knife or gun for protection or fighting”) |  |  |  |
|  |  |  | Weapon use (“used a weapon against another person”) |  |  |  |
|  | **2004 Pelotas birth cohort**^6^ | | | | | |
|  |  | Confidential crime questionnaire, adapted from the Edinburgh Study of Youth Transitions and Crime^5^ | Physical assault (“hit people with the intention of hurting them”) | Person (perpetrator) | 2021 – 2022 (1 year) | Aged 18 years old (past year prevalence) |
|  |  |  | Robbery “made threats or used violence in  robbery”) |  |  |  |
|  |  |  | Weapon carriage (“carried a knife or gun for protection or fighting”) |  |  |  |
|  |  |  | Weapon use (“used a weapon against another person”) |  |  |  |

Data drawn from official records of police- (crimes^2^) and health-recorded violence (mortality^1^, hospitalisations^3^) and self-reports of violence from three consecutive Pelotas birth cohort studies.^4,6,7^ Mortality and hospitalisation by occurrence of incident (e.g., death or hospitalisation), rather than residence.

**Table S2.** Measures of sociodemographic characteristics consistently collected in the 1982, 1993 and 2004 Pelotas Birth Cohorts.

| **Measure** | | **Description** | **Cohort member’s age at measurement** | **Response categories** | **Collapsed response categories** |
| --- | --- | --- | --- | --- | --- |
| **Demographic** | | | | | |
|  | Sex | Child’s sex | Birth | 2 categories:  males vs females | n/a |
|  | Skin colour | Mother’s skin colour: White, and Black/mixed (including Brown with mixed European and African ancestry) | Birth | 2 categories:  White vs  Black/mixed | n/a |
| **Socioeconomic position** | | | | | |
|  | Family income | Based on the family’s household income | Birth | 5 categories: quintiles | 2 categories:  poorest (Q1-Q2) and richest (Q3-Q5) |
|  | Maternal education | The number of years the mother completed of schooling | Birth | 3 categories:  0-4, 5-8, and ≥9y | 2 categories:  low (0-8y) vs high (≥9y) |

Consistently collected sociodemographic measures of self-reported violence across the 1982, 1993, and 2004 Pelotas birth cohort studies, measures identified based on theory and handled in line with prior cross-cohort analyses.^8^ Q, quintiles; y, years.

**Figure S1.** Timeline of self-reported violent crime data in the 1982, 1993 and 2004 Pelotas Birth Cohorts.

*Representation of available data on self-reported perpetration of violence across the 1982, 1993, and 2004 Pelotas birth cohort studies.^4,6,7^ Each row shows a single data collection time point for a cohort (e.g. the first row shows data collected at age 40 in the 1982 birth cohort), with the relevant data collected regarding past year, and lifetime violence. Dark orange with years bolded represent ages and years of data collection, medium orange represents past year prevalence recall period, and light orange represents life-time (including first & last age at offence).*

Lifetime

in adulthood

Past year

18y

**Figure S2.** Overlapping and well-matched measures of self-reported violence in the 1982, 1993 and 2004 Pelotas Birth Cohorts.

*Representation of overlapping measures of self-reported violence across the 1982, 1993, and 2004 Pelotas birth cohort studies.^4,6,7^ Lifetime in adulthood is similar but not identical for the 1982 and 1993 Pelotas birth cohorts as were measured at aged 30 years and 40 years old, respectively.*

**Figure S3.** Homicide victimization rates per 100,000 person-years in Brazil, State of Rio Grande do Sul, and Municipality of Pelotas, 1980-2023.

*Homicide defined as external causes by another person of death (assault, ICD-10: X85-Y09) collected by the national mortality information system (Sistema de Informação sobre Mortalidade, SIM),^1^ 1980-2023.* *Trends were smoothed using LOESS regression with ribbons reflecting 95% confidence intervals.*

**Figure S4.** Official records of violence rates per 100,000 person-years in Pelotas, 1980-2024.

*Trends were smoothed using* *LOESS regression* *with grey ribbons reflecting 95% confidence intervals.*

**Figure S5**. Self-reported prevalence of violence perpetration at matched age windows of year of last offence (0-17, 18-22, 23-30, 31-40 years) in the 1982 and 1993 Pelotas Birth Cohort Studies.

*Self-report measures capture last year of offence prevalence in the 1982 and 1993 Pelotas Birth Cohort Studies. See Table S1 and Figure S2 for full details on the cohort measures****.*** *Cohort prevalence reflects the percent (or rate per 100 persons) of complete cases in each cohort who responded ‘yes’ to violence perpetration.*

**Supplemental references**

1.	Ministério da Saúde. Sistema de Informações Sobre Mortalidade (SIM) [Internet]. Sistema de Informações Sobre Mortalidade (SIM). [cited 2021 Nov 16]. Available from: https://www.gov.br/saude/pt-br/composicao/vigilancia-em-saude-svs/sistemas-de-informacao/sistema-de-informacoes-sobre-mortalidade-sim

2.	Secretaria da Segurança Pública (SSP). Indicadores Criminais [Internet]. Estatísticas. [cited 2025 Sept 24]. Available from: https://www.ssp.rs.gov.br/estatisticas

3.	Ministério da Saúde. Doenças e Agravos de Notificação – 2007 em diante (SINAN) [Internet]. 2023 [cited 2023 Mar 20]. Available from: https://datasus.saude.gov.br/acesso-a-informacao/doencas-e-agravos-de-notificacao-de-2007-em-diante-sinan/

4.	Horta BL, Gigante DP, Gonçalves H, Motta J dos S, Mola CL de, Oliveira IO, et al. Cohort Profile Update: The 1982 Pelotas (Brazil) Birth Cohort Study. Int J Epidemiol. 2015 Mar 2;44(2):441.

5.	McAra L, McVie S. Youth crime and justice: Key messages from the Edinburgh Study of Youth Transitions and Crime. Criminol Crim Justice. 2010;10(2):179–209.

6.	Gonçalves H, Wehrmeister FC, Assunção MCF, Tovo-Rodrigues L, de Oliveira IO, Murray J, et al. Cohort Profile Update: The 1993 Pelotas (Brazil) Birth Cohort follow-up at 22 years. Int J Epidemiol. 2018 Oct;47(5):1389–1390e.

7.	Tovo-Rodrigues L, Santos IS, Bierhals IO, Del-Ponte B, Murray J, Bielemann R, et al. Cohort profile update: 2004 Pelotas (Brazil) Birth Cohort Study follow-up during adolescent years. Int J Epidemiol. 2024;53(1):dyad156.

8.	Degli Esposti M, Matijasevich A, Collishaw S, Martins-Silva T, Santos IS, Menezes AMB, et al. Secular trends and social inequalities in child behavioural problems across three Brazilian cohort studies (1993, 2004 and 2015). Epidemiol Psychiatr Sci. 2023;32:e23.
